# Widespread illegal sales of antibiotics in Chinese pharmacies – a nationwide cross-sectional study

**DOI:** 10.1186/s13756-019-0655-7

**Published:** 2020-01-15

**Authors:** Jie Chen, Yanmei Wang, Xuejie Chen, Therese Hesketh

**Affiliations:** 1 Zhejiang University School of Medicine, 866 Yuhangtang Road, Hangzhou, 310058 People’s Republic of China; 2 Department of Gastroenterology, Third Xiangya Hospital, Central South University, Changsha, 410013 Hunan Province People’s Republic of China; 3 Centre for Global Health, Zhejiang University School of Medicine, 866 Yuhangtang Road, Hangzhou, 310058 People’s Republic of China; 4 Institute for Global Health, University College London, 30 Guilford St, London, WC1N1EH UK

**Keywords:** Anti-microbial resistance, Antibiotic, Anti-microbial stewardship, Pharmacy, China

## Abstract

**Background:**

Access to antibiotics without a prescription from retail pharmacies has been described as a major contributor to anti-microbial resistance (AMR) globally. In the context of high rates of AMR, the Chinese government has recently introduced strict policies regarding hospital antibiotic use, but the existing ban on antibiotic sales without prescription in retail pharmacies has not been strongly enforced. In 2016, a goal of prescription-only antibiotics by 2020 was announced. The objective of the study was to determine progress towards the 2020 goal, through estimating the proportion of retail pharmacies selling antibiotics without prescription across the three regions of mainland China.

**Methods:**

Using the Simulated Patient method, we conducted a cross-sectional survey across purposively-sampled retail pharmacies in urban and rural areas of 13 provinces in eastern, central and western China. Medical students presented a scenario of a mild upper respiratory tract infection, following a strict three-step protocol. They recorded the pharmacy characteristics, and details of their experience, including at which step antibiotics were offered.

**Results:**

Complete data were obtained from 1106 pharmacies. Antibiotics were obtained in 925 (83.6, 95% CI: 81.5, 85.8%) pharmacies without a prescription, 279 (25.2%) at Stage 1 (symptoms only described), 576 (52.1%) at stage 2 (asked for antibiotics), and 70 (6.3%) at Stage 3 (asked for penicillin or cephalosporins). There were significant differences between provinces, with antibiotic access (at any stage) ranging from 57.0% (57/100) in Zhejiang (81/82) to 98.8% in Guizhou. However, there were no significant differences in access to antibiotics by level of city, county, township or village (*P* = 0.25), whether the pharmacy was part of a chain or independent (*P* = 0.23), whether a licensed pharmacist was attending (*P* = 0.82) or whether there was a sign saying that prescriptions were required for antibiotics (*P* = 0.19).

**Conclusions:**

It is easy to obtain antibiotics without a prescription in retail pharmacies in China, despite the fact it is against the law. This must be addressed as part of the wider anti-microbial stewardship effort which could include intense enforcement of the existing law, supported by a public education campaign.

## Background

Anti-microbial resistance (AMR) is acknowledged as one of the greatest threats to global health this century, as well as a major contributor to rising healthcare costs worldwide [1]. It is now a problem in all regions of the world [2]. Mortality attributable to AMR is predicted to rise from 700,000 in 2015, to as high as 10 million by 2050, unless effective control measures are introduced [3].

Misuse of antibiotics, both in medicine and agriculture, is well-established as the major driver of AMR [4]. In medicine, despite awareness by doctors that antibiotics should be used with care, defensive medicine and profit motives are driving the increase in antibiotic use in many countries [5]. A recent study across 76 countries reported a 65% increase in antibiotic use between 2000 and 2015. Most of this increase was in low- and middle-income countries (LMICs), where it was correlated with growth in per capita gross domestic product. Based on this trajectory, global antibiotic consumption will double between 2015 and 2030 [6]. Reducing global consumption is thus crucial to reducing the threat of AMR [6, 7].

Antibiotic stewardship programmes to reduce prescribing of antibiotics by doctors, have been introduced in many countries, especially in hospital settings, with some success in reducing antibiotic misuse [8]. But it is estimated that more than 50% of antibiotics worldwide are purchased without a prescription, from pharmacies, market stalls or street vendors, especially in LMICs [9]. This occurs because of the absence of prescription-only regulations, or lack of enforcement where such regulations do exist. This leads to large quantities of antibiotics in circulation which contribute considerably to AMR [10].

Rising levels of AMR in China are contributing to overall global increases of AMR [11]. In a national survey, 60% of isolates of some species were drug-resistant, including methicillin-resistant *Staphylococcus aureus*, β-lactamase-producing *Escherichia coli*, quinolone-resistant E coli, and carbapenem-resistant *Pseudomonas aeruginosa* [10, 12] In 2016 colistin resistance was reported for the first time in China [13].

This has led to action by the Chinese government. AMR-targeted policies included the banning of antibiotics sales without prescription as early as 2004 [14]. In 2011, the Ministry of Health set up a special task force on antibiotic stewardship, resulting in strict rulings covering all aspects of antibiotic use in hospitals [15]. As a result, the use of antibiotics in many hospitals, especially in tertiary settings, has reduced. However, the use of antibiotics in primary care remains high [16]. Sale of antibiotic in retail pharmacies were not addressed in the 2011 regulations, despite the fact that ease of access to antibiotics without a prescription had been documented [15]. Two studies conducted in 2015 illustrated the ease of access to antibiotics without prescription: the first in pharmacies in three Chinese cities [17], and the second among university students, frequently self-medicating with over-the-counter antibiotics [18]. At the G20 Summit in China in 2016, a comprehensive plan to address AMR was announced, and this included a prominent goal of prescription-only antibiotics at pharmacies in all provinces by 2020 [19]. However, guidance about mechanisms for achieving the goal was notably absent.

The main objective of this study, therefore, was to determine the degree of progress which has been made towards the 2020 goal. We aimed to quantify the proportion of pharmacies where antibiotics could be purchased without a prescription, across the three regions of China. Secondary objectives were to determine the impact of the pharmacies’ geographical location and characteristics, as well as the standard of pharmacy services during the sale of antibiotics.

## Methods

We conducted a cross-sectional survey in 13 provinces, representing all three regions of China: four in the east (Jiangsu, Zhejiang, Fujian and Guangdong), five in the central region (Anhui, Jiangxi, Henan, Hubei and Hunan) and four in the west (Sichuan, Guizhou, Shaanxi and Chongqing). The 13 provinces also represent the range of socio-economic development in China. Sampling of pharmacies was purposive, based on the need for broad representation of different pharmacy characteristics:

1. administrative level, that is, at city, county and township/village level representing the continuum between urban and rural: city is urban, township and village are rural, and the county level, while defined as rural, has mainly urban characteristics. The hypothesis was that access to antibiotics would be easier in rural areas, where enforcement is more difficult.
2. part of a pharmacy chain or independent. We hypothesised that chain pharmacies, some with branches across the country, would be more likely to comply with prescription-only regulations.
3. at a range of distance from a hospital, defined as closer or further than 2 km. Our hypothesis was that pharmacies close to hospitals (which provide most of the primary care in China) would receive more prescriptions for antibiotics and would therefore be more likely to refuse requests without prescription.

The sampling process of pharmacies was multi-stage. We selected the capital city, one small city and one county in each province, and selected a nodal point in each location from which to sample the pharmacies. The aim was to include at least 80 pharmacies in each of the 13 provinces, with an equal number of pharmacies across the three characteristic categories listed above. To achieve this we used the Chinese equivalent of Google maps, Baidu maps, which includes detail of the locations of pharmacies and hospitals, together with the names of the pharmacies, thus identifying whether they were chain or independent. We then selected 35 pharmacies within a 10 km radius of each of the three nodal points in each province. The over-sampling was to allow for possible errors in Baidu maps, and for closures at the time the pharmacies were visited. The distance of 10 km allowed for inclusion of rural pharmacies.

### Procedures

We used the Simulated Patient method to conduct the survey. A simulated patient is an individual trained to act as a real patient in order to simulate a set of symptoms or problems [ 20]. This method has been widely used for research into health care provision in a number of countries, including to determine access to prescription-only drugs in pharmacies [21, 22].

**Fig. 1:**
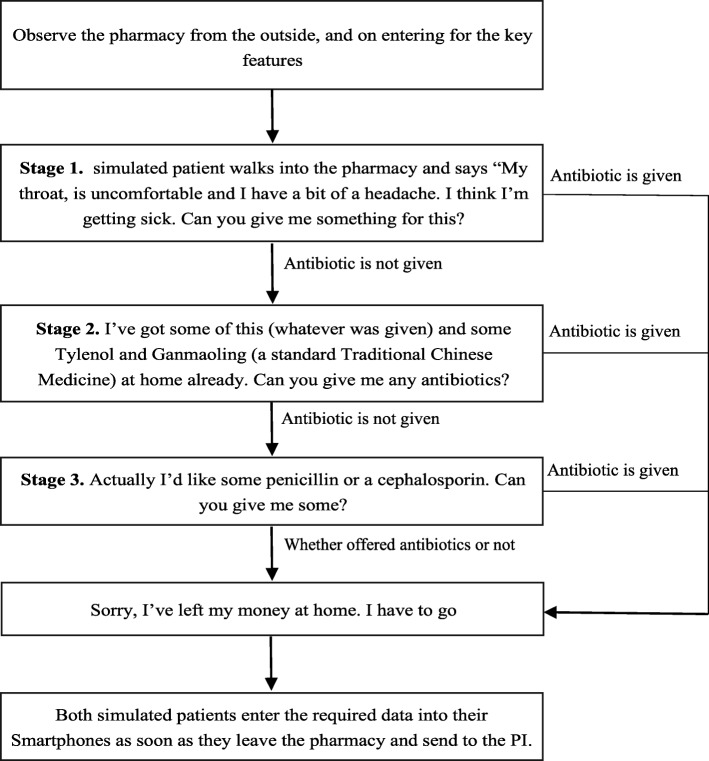
Flowchart of protocol for simulated patient visits

Our simulated patients were undergraduate medical students, from Zhejiang and Xiamen Universities. A notice explaining the study was disseminated on campus via social media, and 40 students were selected at interview, allowing for the allocation of three in each province. The case scenario, shown in Fig. 1, was deliberately chosen to represent a situation where there could be no justification for the use of antibiotics: a description of mild upper respiratory tract symptoms, without visible symptoms, in a healthy young adult. The staged approach, is well-established within the simulated patient methodology, and the exact steps were adapted from research by others [17, 22–24]. All students underwent training in the steps of the protocol. They initially practised with each other, under the observation of the investigators. When they were ready, they were required to conduct a pilot in at least two local pharmacies, to ensure competence with the process and with the reporting requirements, before leaving for their respective provinces.

The data collection was carried out from July to September 2017. Students worked in pairs, taking it in turns to act as simulated patients or to observe and ensure adherence to the protocol. Both independently recorded the findings. All the simulated patients were told they should not act any symptoms, should give no impression of appearing unwell, and that they should not be too insistent about obtaining antibiotics, that is they should only ask once for antibiotics. They were instructed how to respond to likely questions from the pharmacist, that is, they had no other symptoms, except a slight runny nose, and they had no fever. If offered medications, simulated patients were told to say they had left their wallet or purse at home. Therefore, no medications were actually purchased.

The simulated patient pairs were required to enter the following data into a standard form on their Smartphones, as soon as they left the pharmacy, and to send to the principle investigator(PI) immediately:

1. Details of the pharmacy included:
  - the location;
  - a chain or independent;
  - distance from nearest hospital;
  - whether there was a special counter for antibiotics. This is partly a marketing ploy, a place where antibiotics are displayed separately;
  - whether there was a prescription drug sales logo. This is required under legislation, and is a declaration that “prescription-only” drugs, including antibiotics, will not be sold without a prescription;
  - whether the pharmacy had a licensed pharmacist and whether present at the time. This information was obtained from the required licensed pharmacist certificate on the wall and the identification badges worn by the staff.
2. If antibiotics were offered and at which stage (1,2 or 3).
3. Which antibiotics were offered.
4. Whether the pharmacist asked about a) the symptoms, b) whether a doctor had been consulted, and c) allergies, before offering antibiotics.
5. Any other observations of note, especially in relation to the pharmacist’s communication.

## Analysis

Descriptive statistics were reported as frequencies with 95% confidence intervals. Chi-Squared tests were used to compare categorical variables. Analysis was carried-out using SPSS 24.0.

## Results

### Characteristics of the pharmacies

Pharmacy characteristics are shown in Tables 1 and 2. A total of 1345 pharmacies were visited; 239 (17.7%) of the submitted forms had to be discarded, because of incomplete data and/or unacceptable variation from the protocol. Complete information was collected from 1106 pharmacies, 364 (32.9%) from the eastern region, 416 (37.6%) the central region and 326 (29.5%) the west; 367 (33.2%) were at city level 433 (39.2%) at county level, and 306 (27.6%) in townships. There were 702 (63.5%) chain pharmacies, and 588 (53.2%) were located within 2 km of hospitals; 1011 (91.4%) of pharmacies displayed prescription-only drug sales notices, and 922 (83.4%) had antibiotic counters. Just over two-thirds 760 (68.8%) employed a licensed pharmacist. At the time of the simulated patients’ visit 485 (43.9%) were on duty.

**Table 1:** Antibiotics given without prescription at Stages 1, 2 & 3 by province & region

	n(%)	Overall Success n(%) with 95% CI	Success at Stage 1 n (%) with 95% CI	Success at Stage 2 n n(%) 95% CI	Success at Stage 3 n (%) 95% CI
Total	1106(100.0)	83.6 (81.5,85.8).	30.2(27.2,33.1)	62.3(59.1,65.4)	7.6(5.9,9.3)
Eastern					
Zhejiang	100(9.0)	57.0(47.1,66.9)	33.3(20.7,46.0)	59.6(46.5,72.8)	7.0(0.2,13.9)
Jiangsu	92(8.3)	92.4(86.9,97.9)	42.4(31.6,53.1)	52.9(42.1,63.8)	4.7(0.1,9.3)
Fujian	92(8.3)	91.3(85.4,97.2)	31.0(20.9,41.0)	61.9(51.3,72.5)	7.1(1.5,12.8)
Guangdong	80(7.2)	58.8(47.7,69.8)	23.4(10.8,36.0)	68.1(54.3,81.9)	8.5(0.2,16.8)
Central					
Anhui	92(8.3)	95.7(91.4,99.9)	36.4(26.1,46.6)	55.7(45.1,66.3)	8.0(2.2,13.7)
Jiangxi	81(7.3)	97.5(91.4,99.3)	30.4(20.0,40.7)	65.8(55.1,76.5)	3.9(1.4,11.0)
Hunan	83(7.5)	86.7(79.3,94.2)	29.2(18.4,39.9)	63.9(52.5,75.3)	6.9(0.9,13.0)
Henan	80(7.2)	95.0(90.1,99.9)	6.6 (0.9,12.3)	81.6(72.7,90.5)	11.8(4.4,19.3)
Hubei	80(7.2)	72.5(62.5,82.5)	13.8(4.6,22.9)	69.0(56.7,81.2)	17.2(7.2,27.3)
Western					
Sichuan	81(7.3)	72.8(62.9,82.7)	15.3(5.8,24.7)	71.2(59.3,83.1)	13.6(4.6,22.6)
Guizhou	82(7.4)	98.8(93.4,99.8)	34.6(24.0,45.1)	65.4(54.9,76.0)	0
Shanxi	83(7.5)	75.9(66.5,85.3)	38.1(25.8,50.4)	58.7(46.2,71.2)	3.3(0.9,11.2)
Chongqing	80(7.2)	95.0(90.1,99.9)	47.4(35.9,58.9)	42.1(30.7,53.5)	10.5(3.5,17.6)
Region					
Eastern	364(32.9)	75.0(70.5,79.5)	33.7(28.1,39.3)	59.7(53.9,65.6)	6.6(3.6,9.6)
Middle	416(37.6)	89.7(86.7,92.6)	24.1(19.8,28.5)	66.8(62.0,71.6)	9.1(6.2,12.0)
Western	326(29.5)	85.6(81.7,89.4)	34.8(29.1,40.4)	58.8(53.0,64.6)	6.5(3.6,9.4)
*p*-value		0.00	0.004	0.07	0.34

**Table 2:** Antibiotics given without prescription at Stages 1, 2 and 3 by pharmacy characteristics

	n(%)	Overall Success n(%) with 95% CI	Success at Stage 1 n (%) with 95% CI	Success at Stage 2 n (%) 95% CI	Success at Stage 3 n (%) 95% CI
Level					
City	367(33.2)	82.3(78.4,86.2)	23.2(18.8,27.5)	51.2(46.1,56.4)	7.9(5.1,10.7)
County	433(39.2)	82.7(79.1,86.3)	22.6(18.7,26.6)	53.8(49.1,58.5)	6.2(3.9,8.5)
Township	306(27.7)	86.6(82.8,90.4)	31.4(26.1,36.6)	50.7(45.0,56.3)	4.6(2.2,6.9)
Chain	702(63.5)	82.6(79.8,85.4)	23.6(20.5,26.8)	52.0(48.3,55.7)	7.0(5.1,8.9)
Independent	404(36.5)	85.4(81.9,88.9)	28.0(23.6,32.4)	52.2(47.3,57.1)	5.2(3.0,7.4)
*p*-value		0.23	0.11	0.94	0.24
< 2 km from hospital	588(53.2)	81.3(78.1,84.5)	23.6(20.2,27.1)	51.0(47.0,55.1)	6.6(4.6,8.6)
> 2 km from hospital	518(46.8)	86.3(83.3,89.3)	27.0(23.2,30.9)	53.3(49.0,57.6)	6.0(3.9,8.0)
*p*-value		0.02	0.20	0.45	0.66
Special counter for antibiotics	922(83.4)	84.8(82.5,87.1)	24.3(21.5,27.1)	53.9(50.7,57.1)	6.6(5.0,8.2)
No counter for antibiotics	184(16.6)	77.7(71.6,83.8)	29.9(23.2,36.6)	42.9(35.7,50.2)	4.9(1.7,8.0)
*p*-value		0.02	0.22	0.01	0.38
Sign for prescription drugs	1011(91.4)	83.2(80.9,85.5)	24.9(22.3,27.6)	52.3(49.2,55.4)	5.9(4.5,7.4)
No sign for prescription drugs	95(8.6)	88.4(81.9,95.0)	28.4(19.2,37.7)	49.5(39.2,59.7)	10.5(4.2,16.8)
*p*-value		0.19	0.57	0.59	0.08
Licensed pharmacist employed in pharmacy	485(43.9)	80.8(77.3,84.3)	21.9(18.2,25.5)	50.9(46.5,55.4)	8.0(5.6,10.5)
Licensed pharmacist employed, not in pharmacy	275(24.9)	81.1(76.4,85.7)	24.0(18.9,29.1)	51.6(45.7,57.6)	5.5(2.8,8.2)
No licensed pharmacist employed	346(31.3)	89.6(86.4,92.8)	30.9(26.0,35.8)	54.0(48.8,59.3)	4.6(2.4,6.8)
*p*-value		0.001	0.01	0.67	0.11

### Antibiotics sales

Antibiotics were obtained in 925 (83.6, 95% CI: 81.5, 85.8%) pharmacies without a prescription, 279 (25.2%) at Stage 1 (symptoms only described), 576 (52.1%) at stage 2 (asked for antibiotics), and 70 (6.3%) at Stage 3 (asked for penicillin or cephalosporin). This total excluded 15 (1.4%), who said they were willing to provide prescriptions via an on-line consultation and e-prescription to patients who had no prescriptions. Nearly all of these were in the provinces of Sichuan (*n* = 7) and Hubei (*n* = 5).

Of the 181 (16.4%) pharmacies where antibiotics were not offered, the reasons given were: that a prescription was necessary in 113 (10.2%), that antibiotics were not indicated in 58 (5.2%), or that there were no antibiotics in stock in 6 (0.5%).

The antibiotics offered were mainly of three types: penicillins (333/925, 36.0%), cephalosporins (274/925, 29.6%) and macrolides (250/925, 27.0%). The remainder were quinolones, metronidazole and clindamycin. In six cases two antibiotics were offered.

Table 1 shows the “success” rates of acquiring antibiotics by province and region, as well as by the stage at which they were offered. In total, in seven provinces over 90% of the simulated patients were offered antibiotics, in four 70–90%, and less than 70% in just two. The range was from 57.0% in Zhejiang to 98.8% in Guizhou, where there was just one refusal. There were significant regional differences with access easiest in the central region, and hardest in the east (*P* < 0.0001). In the west it was significantly easier to get antibiotics at the first stage (*P* = 0.004), but it was easier to get antibiotics at the second stage in the central region (*P* = 0.07). In total 92.4% of all antibiotics were offered at Stage 1 or 2.

Table 2 shows the success of acquiring antibiotics by pharmacy characteristics. There were no significant differences in access to antibiotics by urban/rural location (city, county, township/village) (*P* = 0.25), or pharmacy ownership, independent or part of a chain (*P* = 0.23). It was easier to get antibiotics in pharmacies more than 2 km from hospitals (*P* = 0.02). Having a special counter for antibiotics increased the offer of antibiotics (*P* = 0.02), but having a prescription-only sign made no difference (*P* = 0.19). The employment of a licensed pharmacist reduced the offer of antibiotics from 89.6 to 80.9% (*P* = 0.0003), but whether the licensed pharmacist was actually attending at the time of the visit made no difference (*P* = 0.93).

### Pharmacy services

These are shown in Table 3. Overall 65.4%(723/1106) asked about symptoms, 11.9%(132/1106) asked the simulated patient if they had a prescription, 24.4%(270/1106) asked about the history of drug allergy before giving antibiotics, and only 1.0%(11/1106) asked if the simulated patient had seen a doctor. Most (785/1106, 71.0%) offered general health and nutrition advice, including promoting the sale of dietary supplements. The attending licensed pharmacists performed significantly worse on all the above. All the pharmacists offered some form of medication apart from the antibiotics: 628 (56.8%) offered Traditional Chinese Medicine (183 different proprietary brands) most commonly Pudilan (11.3%), and Ganmaoling (8.2%).

**Table 3:** Characteristics of pharmacies and pharmacy services

	Ask about symptoms	Ask about prescription	Ask about allergies	General advice
City	59.7(54.6,64.7)	16.3(12.5,20.2)	24.3(19.8,28.7)	70.8(66.2,75.5)
County	70.4(66.1,74.8)	10.9(7.9,13.8)	22.4(18.5,26.3)	70.4(66.1,74.8)
Township	65.0(59.7,70.4)	8.2(5.1,11.3)	27.5(22.4,32.5)	71.9(66.8,77.0)
*p*-value	0.01	0.003	0.29	0.91
Chain	66.5(63.0,70.0)	13.2(10.7,15.8)	24.2(21.0,27.4)	71.1(67.2,74.9)
Independent	63.4(58.6,68.1)	9.7(6.8,12.5)	24.8(20.5,29.0)	68.8(64.3,73.3)
*p*-value	0.29	0.08	0.84	0.23
No licensed pharmacist employed	63.9(59.6,68.2)	13.8(10.7,16.9)	25.6(21.7,29.5)	71.3(67.3,75.4)
Licensed pharmacist employed not present	70.9(65.5,76.3)	14.2(10.0,18.3)	25.5(20.3,30.6)	74.5(69.4,79.7)
Licensed pharmacist attending	63.0(57.9,68.1)	7.5(4.7,10.3)	22.0(17.6,26.3)	67.6(62.7,72.6)
*p*-value	0.08	0.01	0.44	0.16

## Discussion

While ease of access to antibiotics in retail pharmacies has been previously documented in China [17, 18], our results show that despite the prominently announced goal of prescription-only antibiotics by 2020, there has probably been no progress towards the goal. Indeed the situation may have deteriorated, with our results suggesting greater ease of access to antibiotics than reported in Chang et al’s 2015 urban study which also used an simulated patient design [17]. This naturally raises concerns about the role of pharmacies in overall antibiotic misuse and hence anti-microbial resistance. Our findings are strengthened by the fact that they are consistent in urban and rural areas in all Chinese regions, irrespective of ownership (chain or independent), presence of a licensed pharmacist, a special counter for antibiotics, or a sign for prescription-only drugs. This shows clear failure to enforce the law. Our own search of media reports found only 12 convictions for selling antibiotics between 2008 and 2011, with just minor penalties imposed. This lack of enforcement has led to what appears to be the virtual normalisation of illegal sales of antibiotics.

Our results are perhaps more concerning, given the way that we used the simulated patient methodology. Similar studies, mainly from Europe and the Middle East [21–24], have used actors, or third-party approaches, that is representing someone else, typically a relative, unable to come to the pharmacy because of the illness. Chang et al’s three-city Chinese study, used the latter approach, and elicited offers of antibiotics in 56% of paediatric diarrhoea scenarios, and 78% in young adult respiratory infection scenarios, which included fever and cough, symptoms, explicitly excluded in our study. Our simulated patients described very minor symptoms and were told not to be insistent or aggressive. This direct approach is recognised as stronger in Simulated Patient methodology than using the third party approach [21, 22]. Our findings raise a number of issues:

First, we show the importance of the demand side of the consultation. The majority of pharmacists gave antibiotics at Stage 2, “Could you just give me some antibiotics?”, showing that pharmacy staff respond to the specific demands of patients. It is well-documented that doctors often balance appropriate treatment against patient demands [16, 25]. With reports indicating that around two-thirds of Chinese believe that colds and flu should be treated with antibiotics [18], we show that pharmacists readily respond to demands for antibiotics. The relatively high profit margin on many antibiotics provides considerable supply-side incentive [26].

Second, while we found some significant differences in access to antibiotics according to characteristics of the pharmacies, these differences were small. Enforcement was virtually non-existent at all levels in all regions. Having a prescription-only sign, it appears, was for display only, and usually ignored by pharmacists and customers alike. The antibiotic counter just served to reinforce the concept of antibiotics as a commodity. The only major differences we found were between provinces, with lower offers of antibiotics in Zhejiang and Guangdong, both wealthy, developed eastern provinces. However, this is not the full explanation since in Jiangsu and Fujian (both developed eastern provinces) antibiotics were relatively easy to purchase. Subsequent enquiries among pharmacists (who were not in the original study) suggested that the lower offers in Zhejiang may be due to the effect of the G20 Summit held in Hangzhou, the capital of Zhejiang, in 2016. The general crackdown that surrounded this event included warnings to pharmacies about all non-prescription sales of antibiotics, ahead of the announcement that antibiotic sales without prescription would cease by 2020. We speculate that some of this G20 effect has been sustained.

Third, we raise questions about the role of the pharmacy profession. Nearly a third of our pharmacies did not employ a licensed pharmacist. In 2007 the government announced that all pharmacies must employ a licensed pharmacist. But by the end of 2015 only half did, reflecting the national shortage of pharmacists [26]. But there is also a problem of quality. The stated role of pharmacists is to “ensure the rational and legal sale of drugs” [27]. In our study 81.0% of licensed pharmacists sold antibiotics irrationally and illegally, and their services were of a lower standard than unlicensed pharmacy staff. This is not unique to China. A systematic review of 30 studies from LMICs, identified a range of deficiencies in the quality of licensed pharmacists’ practice, including sales of prescription-only drugs [28]. But in China the government is currently seeking to increase the role of pharmacists to reduce pressures on primary care. Clearly, improved training, quality control measures and inspections must be implemented before the scope of work of pharmacists can be increased [17].

There are limitations to our study. First, we conducted the study across 13 provinces, in the three regions, but the far north and far west were not included, raising questions about national generalisability. Second, we included only 80 to 100 pharmacies per province, but we did sample purposively to include the range of locations and types of pharmacy, and this is a very large study compared with other simulated patient studies in pharmacy settings. Third, we used only one clinical scenario which was of a very minor nature, but it rather served to emphasise how consistently easy it was to get unnecessary antibiotics illegally.

### Policy implications

The anti-microbial stewardship programme is creating a new prescribing paradigm in many hospitals across the country. The pharmacy sector is able to follow its own anti-microbial stewardship programme. China with its top-down approaches, is one of the few countries which can actually address this by combining enforcement of the law, with a campaign to educate the general public. Pharmacists need to be trained to explain to customers why antibiotics are refused, and this needs to be reinforced with a public education campaign.

China has achieved very rapid behaviour change in relation to public health measures in the past. For example, in May 2011 punitive measures were introduced for driving under the influence of alcohol, backed-up by a short period of strict enforcement. Within three months behaviour had changed dramatically, with driving under the influence of alcohol reduced by an average of over 50%, gradually introducing a widespread acceptance of zero tolerance of alcohol for driving across much of China [29].

## Conclusion

The very easy access to antibiotics in retail pharmacies in China needs to be addressed as a matter of urgency. This should be part of the wider anti-microbial stewardship effort. This may require a new approach to policy. More work needs be done to meet the goal of prescription-only antibiotics in pharmacies in China.

## Data Availability

The datasets used and/or analysed during the current study are available from the corresponding author on reasonable request.

## Abbreviations

AMR: Anti-microbial resistance
LMIC: Low- and middle-income country
